# Implementation of iterative metal artifact reduction in the pre-planning-procedure of three-dimensional physical modeling

**DOI:** 10.1186/s41205-017-0013-4

**Published:** 2017-03-31

**Authors:** Roy P. Marcus, Jonathan M. Morris, Jane M. Matsumoto, Amy E. Alexander, Ahmed F. Halaweish, James A Kelly, Joel G. Fletcher, Cynthia H. McCollough, Shuai Leng

**Affiliations:** 1 Department of Radiology, 200 First Street SW, Rochester, MN 55905 USA; 2 Siemens Healthineers, 40 Liberty Blvd., Malvern, PA 19355 USA; 3 Department of Dental Specialties, Mayo Clinic, 200 First Street SW, Rochester, MN 55905 USA

**Keywords:** 3D-printing, Computed Tomography, Metal artifact, Iterative metal artifact reduction

## Abstract

**Background:**

To assess the impact of metal artifact reduction techniques in 3D printing by evaluating image quality and segmentation time in both phantom and patient studies with dental restorations and/or other metal implants. An acrylic denture apparatus (Kilgore Typodent, Kilgore International, Coldwater, MI) was set in a 20 cm water phantom and scanned on a single-source CT scanner with gantry tilting capacity (SOMATOM Edge, Siemens Healthcare, Forchheim, Germany) under 5 scenerios: (1) Baseline acquisition at 120 kV with no gantry tilt, no jaw spacer, (2) acquisition at 140 kV, (3) acquisition with a gantry tilt at 15°, (4) acquisition with a non-radiopaque jaw spacer and (5) acquisition with a jaw spacer and a gantry tilt at 15°. All acquisitions were reconstructed both with and without a dedicated iterative metal artifact reduction algorithm (MAR). Patients referred for a head-and-neck exam were included into the study. Acquisitions were performed on the same scanner with 120 kV and the images were reconstructed with and without iterative MAR. Segmentation was performed on a dedicated workstation (Materialise Interactive Medical Image Control Systems; Materialise NV, Leuven, Belgium) to quantify volume of metal artifact and segmentation time.

**Results:**

In the phantom study, the use of gantry tilt, jaw spacer and increased tube voltage showed no benefit in time or artifact volume reduction. However the jaw spacer allowed easier separation of the upper and lower jaw and a better display of the teeth. The use of dedicated iterative MAR significantly reduced the metal artifact volume and processing time. Same observations were made for the four patients included into the study.

**Conclusion:**

The use of dedicated iterative MAR and jaw spacer substantially reduced metal artifacts in the head-and-neck CT acquisitions, hence allowing a faster 3D segmentation workflow.

## Background

The introduction of three-dimensional physical modeling is evolving as an important tool in the medical field, especially in pre-operative planning of complex surgery procedures, medical training or patient education [1–9]. However printing of the final medical three dimensional model is preceded by a number of laborious steps involving computer assisted segmentation of the structures of interest, as described elsewhere in great details [10]. Metal artifacts commonly seen in computed tomography (CT) images, induced by dental hardware or orthopedic prosthesis, hamper the diagnostic evaluation of radiological images, especially affecting the palatine and root of the tongue in cranio-maxillo-facial and head-and-neck imaging [11–13]. Besides the effect on intracorporal anatomical structures, those artifacts also generate extracorporeal artifact-structures, disturbing the fabrication of the 3D-model, adding additional time-consuming segmentation steps to eliminate those structures. In order to overcome these artifacts, acquisitions and post-processing techniques, such as higher x-ray tube voltage, gantry / head tilting, dual-energy or dedicated metal artifact reduction algorithms have been proposed and implemented in diagnostic CT exams [12, 14–17]. However, the impact of these techniques on segmentation of data for 3D printing has not been thoroughly investigated.

The purpose of this study is to assess the impact of metal artifact reduction techniques in 3D printing by evaluating image quality and segmentation time in both phantom and patient studies with dental restorations and/or other metal implants.

## Methods

### Scanner description and data acquisition

All phantom and patient scans were performed on a 128 slice single-source computed tomography (CT) scanner (SOMATOM Definition Edge, Siemens Healthcare, Forchheim, Germany). Imaging parameters of standard clinical protocols are displayed in Table 1.

**Table 1:** Baseline imaging parameters used for the phantom and patient studies

	Phantom study	Patient study
Scan type	Spiral	Spiral
Collimation [mm]	128 x 0.6	128 x 0.6
Tube potential [kV_p_]	120	120
Pitch	0.6	0.8
Rotation time [s]	1	1
Quality reference mAS	135	320
Reconstruction slice thickness [mm] / increment [mm]	0.6 / 0.6	0.75/0.7
Reconstruction kernel	J40 (iterative reconstruction with a strength of 3)	J40 (iterative reconstruction with a strength of 3)

### Phantom studies

**Fig. 1:**
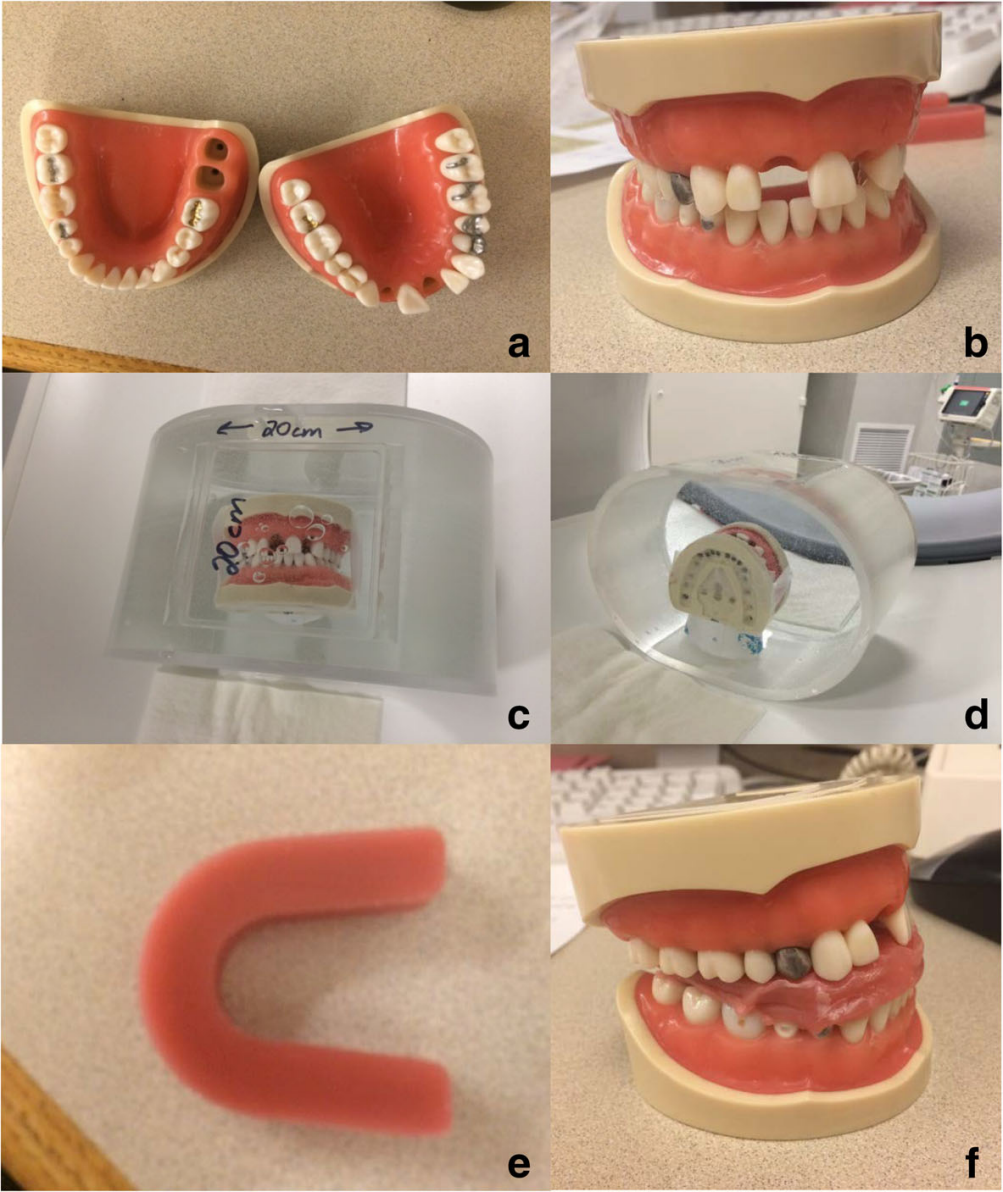
Artificial denture (**a** and **b**) placed in a 20 cm water phantom and (**c** and **d**) used for the phantom study with display of a jaw spacer (**e** and **f**)

A plastic dental apparatus model (Kilgore Typodent, Kilgore International, St. Coldwater, MI) with artificial teeth and randomly distributed dental restorations (*n* = 8) was used in the phantom study, which was placed in a 20 cm water phantom in order to mimic the attenuation of a head (Fig. 1a-d). To investigate different metal artifact reduction techniques, the jaw model was scanned with the following scenarios:

1. Baseline acquisition with imaging parameters as standard clinical protocols (Table 1)
2. Increased tube voltage from 120 to 140 kV while maintaining the remaining baseline acquisition parameters the same.
3. Tilting the gantry by 15° while maintaining the remaining baseline acquisition parameters the same.
4. Insertion of a jaw spacer (Bite Block, Heraeus Kulzer, South Bend, IN), made out of a non-radiopaque material, between the jaw bones (Fig. 1e-f) and acquisition with baseline parameters.
5. Inserting a jaw spacer and acquiring in tilted gantry position of 15° with baseline parameters.

All images were reconstructed using an iterative reconstruction algorithm (ADMIRE, Siemens Healthcare, Forchheim, Germany) and a medium sharp kernel (J40). In addition, all images were reconstructed using a dedicated iterative metal artifact reduction algorithm (MAR) (iMAR^®^, Siemens Healthcare, Forchheim, Germany).

### Patient study

The retrospective patient study was approved by the local institutional review board and was compliant with the Health Insurance Portability and Accountability Act (HIPAA). Informed consent was waivered. Four patients referred for a head and neck acquisition with subsequent 3D-modelling were identified in the institutional database and included into the study. Scan parameters applied are described in Table 1. All images were reconstructed with and without dedicated MAR algorithm.

### Qualitative and quantitative evaluation

Qualitative visual evaluation of the obtained images regarding metal artifacts was performed on a dedicated workstation (snygo.via VB10, Siemens Healthcare, Forchheim, Germany) by a radiologist with >4 years of radiological experience (R.P.M.), focusing on the amount of metal artifacts and how they affect surrounding anatomies.

Quantitative evaluation of the metal artifacts for all above mentioned scenarios was performed using dedicated medical 3D-printing software (Materialise Interactive Medical Image Control Systems; Materialise NV, Leuven, Belgium) by one radiologist with >4 years (R.P.M) of radiological experience. Segmentation of the metal artifact and skull was performed using the split mask tool (Materialise Interactive Medical Image Control Systems; Materialise NV, Leuven, Belgium). The segmented model, including the segmented artifact, was then loaded into 3-matic software (Materialise Interactive Medical Image Control Systems; Materialise NV, Leuven, Belgium), where the volume of the isolated rendered metal artifact was isolated and calculated.

## Results

### Phantom studies

#### Tube voltage and iterative based metal artifact reduction technique – scenarios 1 & 2

**Fig. 2:**
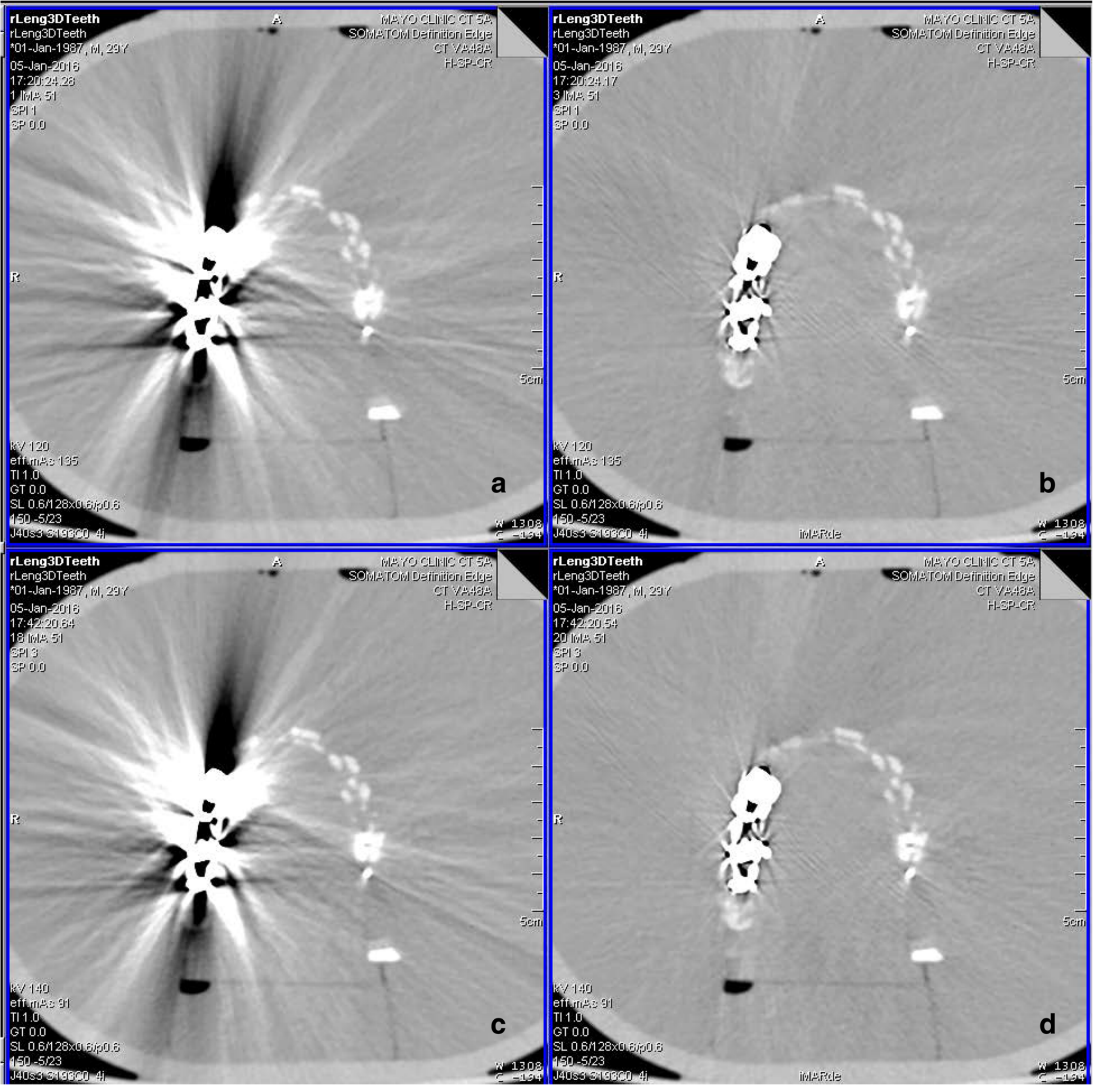
Images of denture phantom scanned at 120 kV (**a**) and 140 kV (**c**), showing no difference in metal artifact. The use of dedicated metal artifact reduction algorithm extensively decreased the artifacts at both 120 kV (**b**) and 140 kV (**d**)

Phantom acquired at 120 kV showed extensive intra- and extracorporal metal- and beam hardening artifacts, comprehensively impairing the visualization of the anatomical structures (Fig. 2a). Adding MAR in the reconstruction process eliminated the majority of intra- and extracorporal metal- and beam hardening artifacts, resulting in a better delineation of the neighboring structures and the jaw itself (Fig. 2b). The use of MAR reduced the metal artifact volume by 96.6% (from 7.7 ml to 0.26 ml) with a time saving of 40 s (from 260 to 220 s). Increasing tube potential to 140 kV did not decrease any metal artifacts (Fig 2c and d).

#### Gantry tilt – scenario 3

**Fig. 3:**
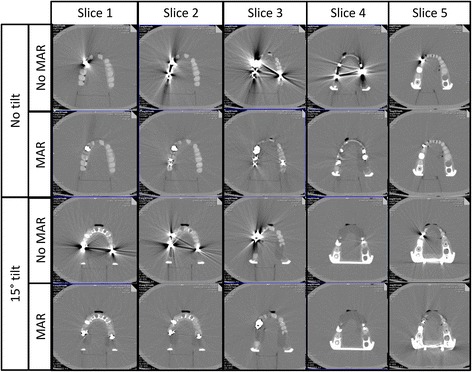
Slice series of the phantom acquisition with and without gantry tilt. In addition, all series were reconstructed with dedicated metal artifact reduction (MAR)

Tilting the gantry by 15° resulted in image datasets with displaced teeth to the previous or following image slices, when compared to the non-tilted acquisition (Fig. 3). Hence the reduced number of metal implants in the same scan plane was associated with lower artifacts per slice. However, the number of slices with metal artifacts increased. Total artifact volume was similar between the tilted and non-tilted acquisition (6.7 vs. 7.7 ml for tilted vs. non-tilted, respectively) with a difference of 65 s in segmentation time (325 vs. 260 s for tilted vs. non-tilted, respectively) as more slices need to be manually cleaned in the case of gantry tilt. Images processed with MAR in the non-tilted position were shown to have a significantly reduced metal artifact volume, when compared to the tilted position (from 4.4 to 0.26 ml).

#### Jaw spacer – scenario 4

**Fig. 4:**
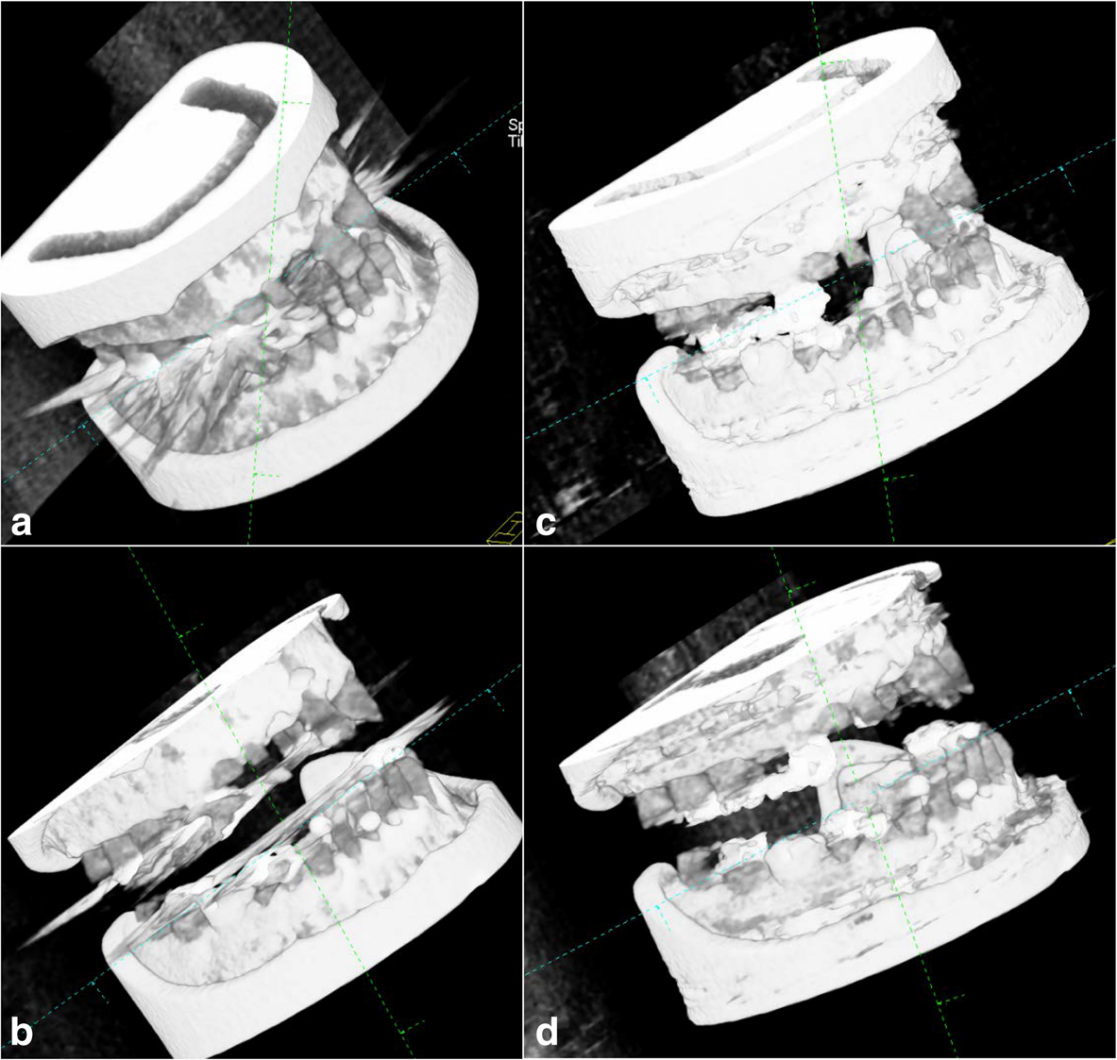
Volume rendering of the denture phantom without jaw spacer and without iterative metal artifact reduction algorithm (**a**). The use of the jaw spacer allowed a perfect separation of the upper from the lower jaw and hence a better separation of the metal artifacts and better depiction of the artificial teeth (**b**). The use of dedicated iterative metal artifact reduction extensively reduced the metal artifacts in the closed jaw phantom (**c**); the combination of jaw spacer and iterative metal artifact reduction allowed a perfect display of the denture with an extensive reduction of metal artifacts (**d**)

The separation of the upper and lower jaw by introducing the spacer allowed a confinement of the artifacts to their respecting plane (Fig. 4a and b). Although no significant difference in artifact volume was identified (6.6 vs. 7.7 ml in with jaw spacer vs. without jaw spacer, respectively), it was easier to separate the maxilla from the mandible with the spacer in place which is often requested clinically in these models. Same observations were made for the images reconstructed using MAR, however with a decreased amount of metal- and beam hardening artifacts (Fig. 4c and d).

#### Combinations of gantry tilt and jaw spacer – scenario 5

**Fig. 5:**
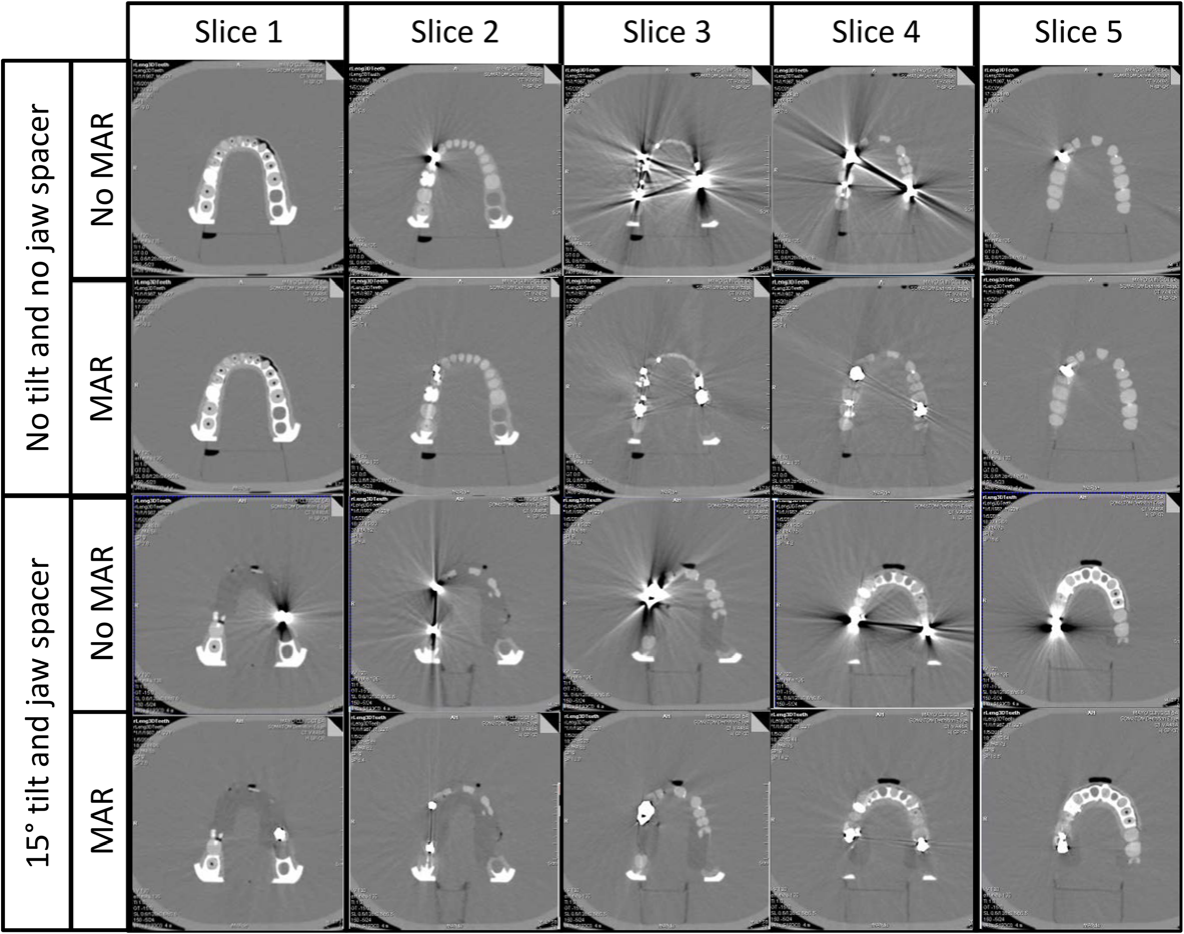
Phantom slice series combining all possible metal artifact reduction techniques

The combination of gantry-tilt and jaw spacer was associated with reduced metal artifact per scan plane and better constraint of the artifacts to the upper and lower jawbones in the images without MAR (4.9 ml) (Fig. 5). The use of MAR significantly reduced the intra- and extracorporal artifact structures (1.8 ml) (Fig. 5).

### Patient study

**Fig. 6:**
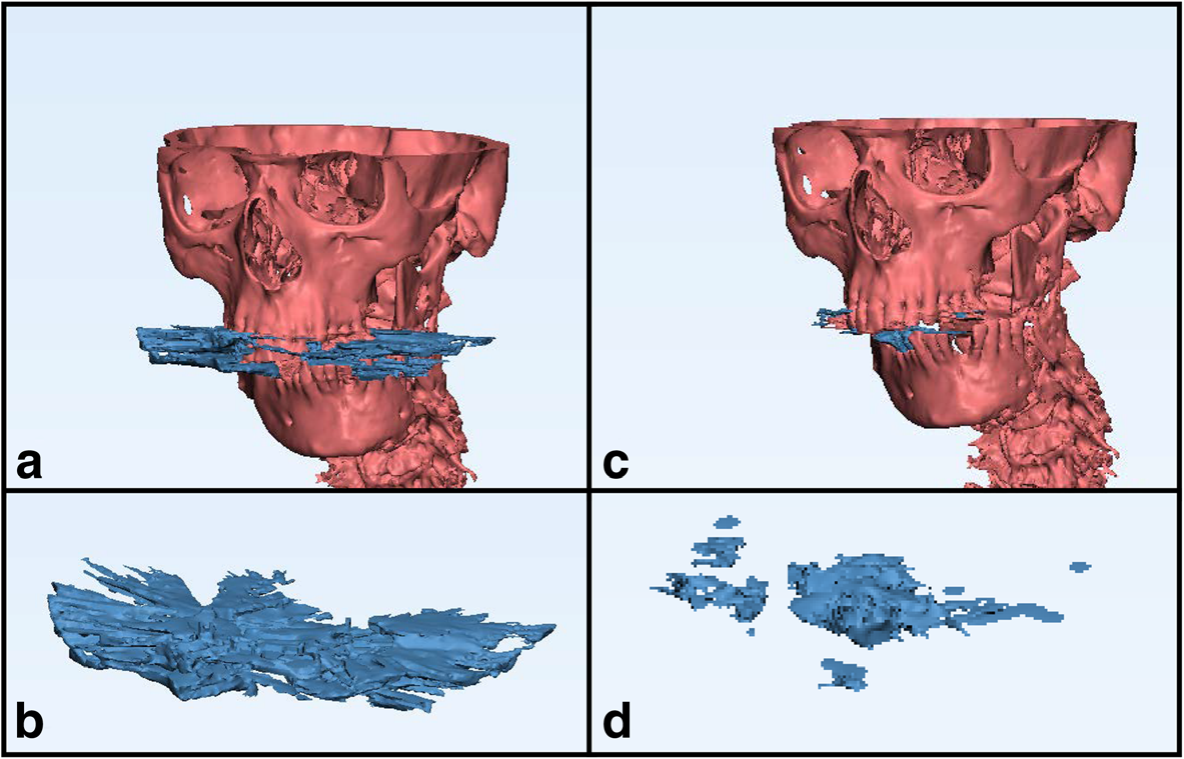
Patient acquired without jaw spacer and no iterative metal artifact reduction algorithm (MAR) showing extensive metal artifact affecting the display of the frontal upper teeth (**a**). The segmented extensive metal artifact had a volume of 36 ml (**b**). The use of MAR (**c**) reduced the metal artifact by 90.8% (**d**). In addition, MAR allowed a better dental display

**Fig. 7:**
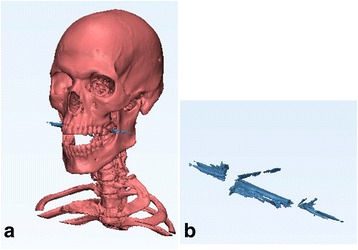
Images of a patient scanned with jaw spacer and reconstructed with dedicated iterative metal artifact reduction algorithm (**a**). The combination of both metal artifact reduction techniques allowed an excellent display of the denture structure with a low metal artifact volume (**b**, 3.4 ml)

Four patients were included in this study. Only one patient was acquired with a jaw spacer, whereas no acquisitions were performed with a tilted gantry. All subjects included into the study were male patients with a mean age of 46 ± 3. Mean artifact volume segmented in the images without dedicated iterative metal artifact reduction was 20.8 ± 15.2 ml with a mean segmentation time of 275 ± 73.7 s (Fig. 6). The use of dedicated iterative MAR over all four patients reduced the mean segmented artifact volume and segmentation time by 79.8% (4.2 ± 0.9 ml) and 40.1% (154 ± 38.6 s), respectively (Table 2). In addition, patient images reconstructed with MAR had a better delineation of the teeth, allowing a better and more natural appearing 3D printing model (Fig. 7). Only one patient with small number of metal dental hardware was acquired with a jaw spacer installed, showing a reduction of artifact volume by 49.3% when reconstructed with the dedicated iterative metal artifact reduction algorithm (3.4 ± 6.7 ml). The use of iterative MAR reduced segmentation time by 67.4% (from 310 to 101 s).

**Table 2:** Artifact Volume and segmentation time for 4 patients referred for head-and-neck acquisition and reconstructed with and without iterative metal artifact reconstruction. Only one patient was acquired with a jaw spacer, whereas none of the acquisitions were performed with a tilted gantry

Patient	Iterative MAR	Artifact Volume [ml]	Segmentation time [s]	Jaw spacer
1	No	36	340	No
	Yes	5.6	200	
2	No	4.5	150	No
	Yes	4.5	135	
3	No	36	300	No
	Yes	3.3	180	
4	No	6.7	310	Yes
	Yes	3.4	101

## Discussion

In this study we demonstrated the use of various metal artifact reduction techniques in order to enhance the anatomical 3D segmentation hampered by heavy metal artifacts. In addition we showed that using dedicated iterative metal artifact reduction algorithm is the most promising technique to reduce the metal artifact volume and hence reducing segmentation time. Those findings are important, since a majority of head-and-neck CT acquisitions are associated with metal artifacts due to dental restorations. This results in laborious 3D segmentation of the craniomaxillofacial bones and separation of the mandible from the maxilla which is needed when 3D printing clinical cases. Previous studies evaluating metal artifact reduction techniques mainly focused on the diagnostic performance and our observations made in the phantom and patient studies agreed with the findings in the present literature [15, 17–19].

Bannas et al. showed an incremental value of gantry tilt on the reader’s sensitivity of detecting oral tumors as gantry tilt redistributed the metal artifacts to other locations outside of the slices where the tumor was [15]. Our phantom data support the visual results, however quantitatively the segmented artifact volume was not different between both gantry states. Artifact segmentation time of the data in non-tilted position was shorter as the artifact did not have to be removed from as many slices. This is mainly due to the concentration of artifact in a smaller and more condensed volume, resulting in a faster and simplified containment. In clinical practice, if the sole purpose of the CT exams is for 3D modeling, patients should be scanned without gantry tilt, as demonstrated by our phantom data. However, if the scan is for both 3D printing and other diagnostic tasks (e.g. tumor detection) where gantry tilt benefits the diagnosis, the patients should be scanned with gantry tilt. In this case, the benefit of accurate tumor detection outweighs the drawback of increased processing time for 3D modeling. Dedicated MAR technique should be used, which has been shown to have a positive impact for the clinical evaluation and 3D-segmentation.

The use of a jaw spacer has been recommended for maxillofacial CT-acquisitions in order to separate both jaws [20]. Our data support the recommendations without having any effects on the segmented artifact volumes. Clinically many of our 3D printed craniomaxillofacial cases are for tumor resection or congenital facial reconstruction and the surgeon often requests the mandible be printed separately from the maxilla for surgical planning. This requires two separate volumes to be created which is hampered by overlapping artifact which is greatly reduced with the jaw spacer. A separate benefit of the spacer was more realistic appearance to the teeth after segmentation. The most time and labor effective artifact reduction method in both phantom and patient model is the dedicated iterative metal artifact reduction [16]. Our study hence adds to the current literature stating that the use of this technique enhances the 3D-segmentation workflow. As demonstrated in a previous study, the MAR algorithm can occasionally deteriorate the bone contours if the parameter settings were not optimal [21]. Therefore, caution should be taken using MAR algorithm and bone contour should be carefully checked based on professional expertise during the segmentation process.

Our study has a number of limitations. First, our patient sample size is very small, since the number of patients referred for pre-operative 3D-printing was limited. Secondly, the phantom used was made of acrylic, which has lower attenuation than bone and consistent CT number unlike the bone which is more variable. In this study, we lowered the CT number threshold in the segmentation process to accommodate this difference between the material properties. This procedure, however, was not necessary in the human subject studies. As seen in our patient study, substantial improvement of image quality was achieved with real bones. Another limitation is that the model after thresholding and manually alteration was used as the reference model when artifact volume was calculated. Since this process was done separately for images reconstructed with and without MAR, it is possible there are slight differences in the artifact volume based on manual artifact removal. Building a common reference model with other techniques, such as 3D scanning, may provide a more accurate estimation of artifact volume. However, we expect the difference would be small as the radiologist tried to maintain the same anatomy after artifact removal and carefully reviewed the final data before calculating artifact volume.

## Conclusion

In conclusion, the use of metal artifact reduction techniques, especially iterative metal artifact reduction algorithm, shortens the time of segmentation for 3D printing and provides a more accurate mandible and maxilla in areas affected by metal artifacts.
